# Early‐Onset Oral Squamous Cell Carcinoma: Emerging Biological Insights, Risk Factors and Clinical Implications

**DOI:** 10.1111/jop.70130

**Published:** 2026-03-15

**Authors:** Gennaro Musella, Cristina D'Antonio, Federica Canfora, Michele D. Mignogna, Valentino Vellone, Vito Carlo Alberto Caponio, Amerigo Giudice, Alessandro Villa, Daniela Adamo

**Affiliations:** ^1^ Department of Clinical and Experimental Medicine University of Foggia Foggia Italy; ^2^ Oral Medicine, Oral Oncology and Dentistry, Miami Cancer Institute Baptist Health South Florida Miami Florida USA; ^3^ Department of Health Sciences, School of Dentistry Magna Graecia University of Catanzaro Catanzaro Italy; ^4^ Department of Neuroscience, Reproductive and Odontostomatological Sciences University of Naples Federico II Naples Italy; ^5^ Department of Life Sciences, Health and Health Professions Link Campus University Rome Italy; ^6^ Department of Orofacial Sciences, School of Dentistry University of California San Francisco San Francisco California USA

**Keywords:** early‐onset oral cancer, NSND EO‐OSCC, oral squamous cell carcinoma, sugar‐sweetened beverages, tumor microenvironment, young adults

## Abstract

**Background:**

Early‐onset oral squamous cell carcinoma (EO‐OSCC), commonly defined as occurring in individuals under 50 years of age, is increasingly recognized as a potentially distinct clinical subset with differences in exposure patterns and tumor biology compared with conventional oral squamous cell carcinoma (OSCC). Unlike typical OSCC, which is strongly associated with tobacco and alcohol, EO‐OSCC more frequently affects never‐smokers/never‐drinkers, posing challenges for early recognition, risk stratification, and management.

**Objective:**

This review integrates evidence mapping with narrative synthesis to summarize current knowledge on EO‐OSCC, focusing on epidemiology, emerging risk factors, molecular alterations, tumor microenvironment characteristics, and clinical implications.

**Methods:**

Literature was searched across PubMed, Scopus, Embase, Web of Science, and the Cochrane Library up to August 2025, complemented by global cancer databases (GLOBOCAN/Cancer Tomorrow) and international trial registries.

**Results:**

Available data suggest distinct biological features, including relatively low tumor mutational burden, recurrent alterations in CDKN2A, TP53, NOTCH1, and EGFR, epigenetic dysregulation, and a checkpoint‐dominant immunosuppressive tumor microenvironment. Alongside traditional carcinogens, modifiable determinants such as sugar‐sweetened beverages, electronic cigarette use, and oral microbial dysbiosis have emerged as plausible contributors, although evidence remains heterogeneous. Despite frequently aggressive histopathologic features, younger patients may tolerate intensive multimodal therapies better and achieve favorable outcomes.

**Conclusion:**

These findings highlight the urgent need for age‐specific prognostic tools, biomarker‐guided therapies, and early detection protocols. International collaboration will be key to improving survival and long‐term quality of life in this growing patient population.

## 1 Introduction

Cancer has traditionally been considered a disease of aging; however, a rising incidence among younger individuals has been reported across several malignancies, including colorectal, breast, gastric, pancreatic, and head and neck cancers. Within this context, early‐onset oral squamous cell carcinoma (EO‐OSCC) has gained increasing attention due to emerging evidence of age‐specific etiologic mechanisms, tumor biology, and clinical behavior compared with conventional oral squamous cell carcinoma (OSCC) [1].

OSCC accounts for approximately 90% of oral malignancies and has historically been associated with cumulative exposure to alcohol and tobacco, predominantly affecting individuals over 50 years of age. In contrast, EO‐OSCC, commonly defined as occurring before 50 years old, represents approximately 4%–10% of cases and shows an increasing incidence, particularly in the oral tongue, although the lack of standardized age cutoffs limits comparability across studies [2].

A substantial proportion of young patients lacks traditional risk exposures, prompting investigation into alternative mechanisms, including germline susceptibility, molecular and epigenetic alterations, oral dysbiosis, and emerging dietary and environmental factors [3, 4, 5]. Accumulating evidence also indicates distinct tumor microenvironment features, supporting EO‐OSCC as a biologically differentiated subtype rather than a simple age‐related variant [6].

Clinically, EO‐OSCC is often perceived as aggressive based on histopathologic features, yet survival outcomes remain heterogeneous. Improved treatment tolerance in younger patients may partially explain preserved or favorable overall survival (OS). Nevertheless, major gaps persist regarding standardized definitions, prognostic biomarkers, and tailored management strategies [7, 8].

This review aims to provide an integrated perspective of EO‐OSCC, addressing epidemiology, risk factors, molecular and immune features, clinical presentation, prognosis, and therapeutic implications.

## 2 Search Strategy and Evidence‐Mapping Approach

This review combines evidence mapping with narrative synthesis to characterize the current research landscape on EO‐OSCC in young adults.

A structured literature search was conducted in PubMed, Scopus, Web of Science, Embase, and the Cochrane Library to identify relevant studies published up to August 2025, using combinations of the terms “early‐onset,” “oral squamous cell carcinoma,” and “young adults.” Epidemiological data were retrieved from global cancer databases (GLOBOCAN and Cancer Tomorrow), and information on ongoing or completed clinical trials was obtained from ClinicalTrials.gov and the WHO International Clinical Trials Registry Platform.

**FIGURE 1:**
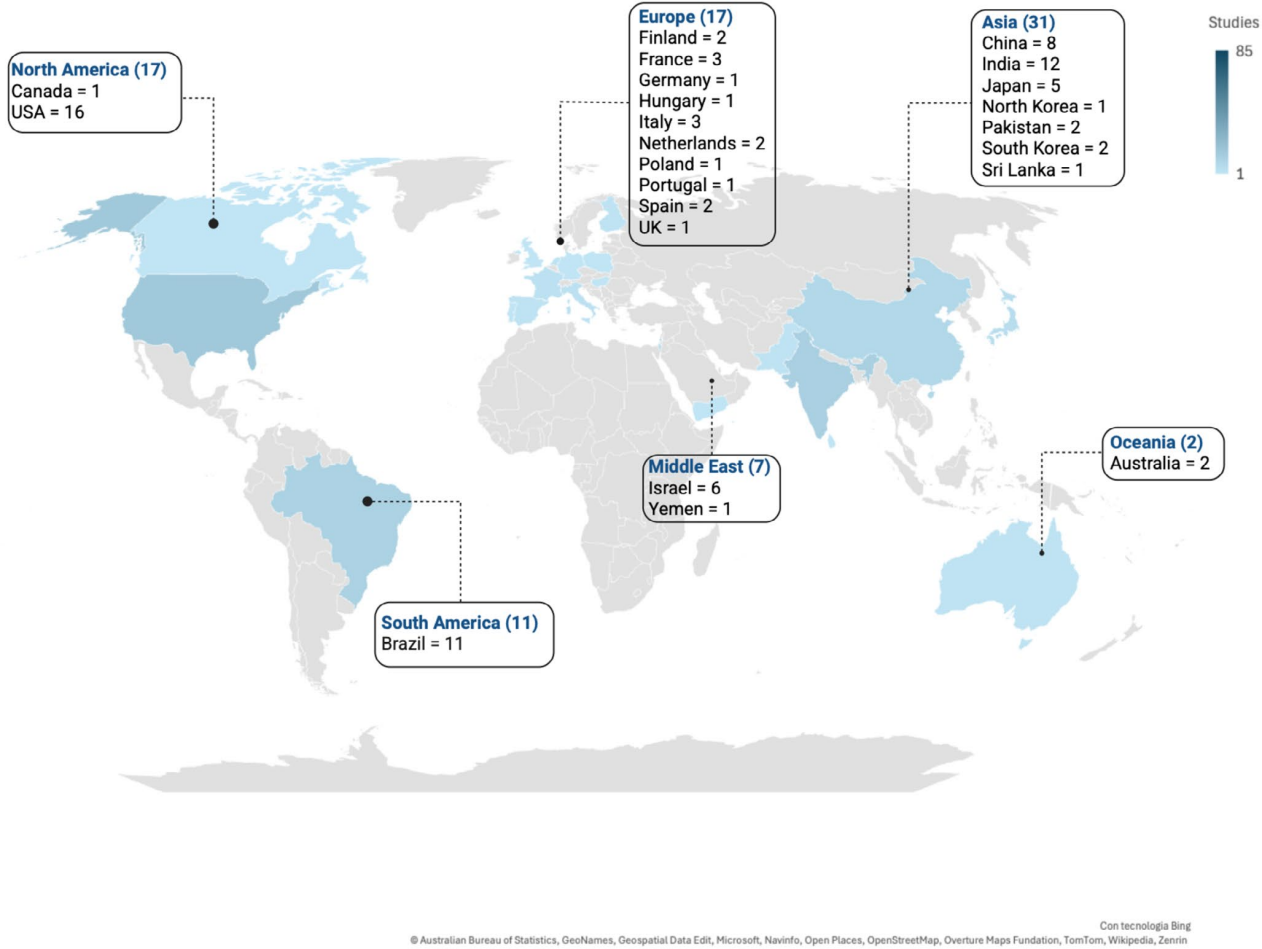
**Evidence mapping of published studies on EO‐OSCC by geographic region.** This figure illustrates the geographic distribution of published studies addressing EO‐OSCC compared to traditional OSCC. Asia accounts for the largest number of studies (31), followed by Europe (17), North America (17), South America (11), the Middle East (7), and Oceania (2). Country‐level study counts are indicated within each region. The complete list of included articles is provided in Table S1‐Supporting Information. This figure reflects the distribution of available published evidence and does not represent population‐based incidence, prevalence, or disease burden. Abbreviations: EO‐OSCC = Early‐Onset Oral Squamous Cell Carcinoma; OSCC = Oral Squamous Cell Carcinoma; USA = United States of America; UK = United Kingdom.

Evidence mapping was applied as a descriptive approach to organize the available literature by geographic distribution and thematic focus, and to visualize research density across key domains (Figure 1; Table S1‐Supporting Information). As this step was exploratory in nature, no meta‐analysis and no formal risk‐of‐bias assessment were performed. Findings were subsequently integrated through narrative synthesis to highlight emerging biological insights, risk patterns, clinical implications, and remaining knowledge gaps.

## 3 Epidemiology and Global Incidence Trends

The epidemiology of EO‐OSCC indicates a relatively low but steadily increasing prevalence over recent decades. According to projections from Cancer Tomorrow (Global Cancer Observatory, WHO/IARC), the global burden of EO‐OSCC is expected to rise globally by 2045, with the largest proportional increases in Africa (~72%) and Oceania (~27%), more moderate growth in Asia, Northern America, and Latin America (6%–11%), and a decline in Europe (~20%) [9].

Population‐based studies consistently report that EO‐OSCC accounts for approximately 4%–10% of OSCC cases, with variability related to age cutoffs and geographic context. A multicenter study including 10 727 OSCC cases across eight referral centers (1998–2018) reported 5.8% of cases in individuals ≤ 40 years [10]. Similarly, a meta‐analysis of 24 studies (age cutoffs 30–50 years) including 46 858 OSCC cases estimated a pooled prevalence of 9.7% for early‐onset disease [2]. Another systematic review and meta‐analysis of 38 studies confirmed persistent male predominance and identified the tongue as the most frequent primary site of EO‐OSCC [3].

Incidence trends among younger populations have increased across multiple regions, although the underlying drivers remain incompletely defined. Human Papillomavirus (HPV) has been proposed as a contributing factor in some populations; however, its etiologic role in EO‐OSCC appears inconsistent and likely dependent on tumor subsite and geographic context [10].

Marked global disparities are evident: based on GLOBOCAN 2022 estimates, Asia accounts for approximately 81% of EO‐OSCC cases worldwide, followed by Europe and Africa (each around 6%), Latin America and the Caribbean (4%), and Northern America (3%), while Oceania contributes less than 1%. These patterns underscore a disproportionate burden in Asian populations and the need for region‐specific prevention strategies [9].

Figure 1 and Table S1‐Supporting Information are retained to illustrate the global distribution of published evidence within an evidence‐mapping framework, while Table 1 summarizes key epidemiological studies, highlighting variability in definitions, populations, and reported incidence trends.

**TABLE 1:** Epidemiological studies reporting incidence trends and demographic characteristics of EO‐OSCC.

N. ^a^	Authors	Year	Age cut‐off	Geographic distribution	Incidence/trends/demographics
1.	Myers et al.	2000	< 40 years	USA	25‐year trend showing increased incidence among young patients, with a male predominance; tongue as the most common site.
2.	Müller et al.	2008	< 40 years	USA	Fourfold increase in OSCC among young individuals since 1974; accounts for 5% of total cases; mobile tongue more frequently affected than in older patients.
3.	Sherin et al.	2008	< 40 years	India (North Kerala)	High incidence in young adults; tongue commonly affected; many cases lack traditional risk factors
4.	Patel et al.	2011	18–44 years	USA	Rising incidence of oral tongue SCC in white individuals aged 18–44; greater increase observed in females; data based on SEER registry.
5.	McGorray et al.	2012	20–44 years	USA	Increasing incidence of tonsillar/base of tongue cancers in white males; decline in oral OSCC; more marked rise in the 45–64 age group.
6.	Tota et al.	2017	< 50 years	USA	Increasing trend in oral tongue cancer (1973–2012); more pronounced rise among younger patients; 1.7% annual increase observed in females.
7.	Al‐Jamaei et al.	2022	< 35 years	Netherlands	Epidemiological analysis of young‐onset OSCC, including age and sex distribution.
8.	Ferreira e Costa et al.	2022	≤ 40 years	India	Among 10 727 OSCC cases, 5.8% occurred in young individuals; Manipal reported 13.2%; tongue and floor of mouth were most frequently involved.
9.	Deneuve et al.	2022	Not specified	France	Significant rise in oral tongue cancer among young French women aged 30–40; divergent trends observed between 1990 and 2018.
10.	Révész et al.	2023	< 40 years	Hungary	56% of head and neck cancers in young patients involved the oral cavity; lower tobacco and alcohol use compared to general head and neck cancers.
11.	Sakr et al.	2023	< 40 years	Egypt	Study of 197 tongue cancer cases (2006–2021); equal gender distribution; significant increase in patients under 40 years.
12.	da Silva Souto et al.	2024	< 40 years	Brazil	Tongue SCC in young patients: 57% were never‐smokers; 43% reported alcohol use; advanced stages prevalent at diagnosis.
13.	Singh et al.	2024	< 45 years	Canada, India, Singapore	Among young patients, 49% presented with tongue involvement; aggressive histopathological features commonly observed.
14.	Sufiawati et al.	2024	Not specified	Indonesia	OSCC accounted for 70% of 627 cases; 37% aged 30–49; tongue affected in 69%; near‐equal male‐to‐female ratio.
15.	Szewczyk et al.	2024	< 45 years	Poland	15.5% of the cohort was younger than 45 years; tongue involved in 45% of cases; majority were male; lower smoking prevalence in younger patients.
16.	Hong et al.	2025	< 40 years	Canada	4.5% of OSCC cases occurred in patients under 40; lower smoking rates; T1 stage more common in younger individuals.

Abbreviations: EO‐OSCC = Early‐Onset Oral Squamous Cell Carcinoma; OSCC = Oral Squamous Cell Carcinoma; SCC = Squamous Cell Carcinoma; SEER = Surveillance, Epidemiology and End Results; USA = United States of America.

^a^ Reference list of the studies is provided in Appendix.

## 4 Defining EO‐OSCC: Age Cutoffs and Conceptual Challenges

Despite increasing attention to EO‐OSCC, no universally accepted age cutoff currently exists to define “early‐onset” disease. Across studies, thresholds range from 30 to 50 years, with most cohorts adopting < 40 or < 45 years, often based on registry conventions rather than biological rationale [1]. Recent investigations have increasingly adopted a < 45‐year cutoff, consistent with definitions used for other early‐onset malignancies [10], while stricter cutoffs (< 40 years) have been proposed to better capture differences in risk factors and tumor biology [11]. Less commonly, registry‐based analyses extend the upper age limit to 49 years, aligning EO‐OSCC with broader classifications of young adult cancers [12].

This heterogeneity is well illustrated by a 2021 meta‐analysis, in which approximately 90% of included studies defined young patients as ≤ 40 years, 6.7% used ≤ 45 years, and only a minority adopted narrower or broader criteria [2]. Such variability hampers cross‐study comparability, complicates pooled analyses, and may contribute to inconsistent conclusions regarding clinical behavior and prognosis.

Importantly, chronological age alone may be insufficient to capture the biological complexity of EO‐OSCC. Integrating age with clinical features, molecular alterations, and exposure history may provide a more informative framework, in line with precision oncology principles.

Nevertheless, stratifying EO‐OSCC by standardized age categories, rather than relying on a single arbitrary threshold, may improve reproducibility and interpretability across studies. Until standardized criteria are established, studies should clearly justify their chosen cutoff and adopt consistent stratification methods.

Key clinical and biological differences between EO‐OSCC and conventional OSCC are summarized in Table 2.

**TABLE 2:** Main differences between EO‐OSCC and conventional OSCC.

	Early‐onset OSCC	Traditional OSCC
Epidemiology	Accounts for 5%–10% of all OSCC cases; incidence increasing globally, particularly among young females and in parts of Asia and Europe.	Represents most cases; incidence stable or declining in high‐income countries.
Sex distribution	Increasing female prevalence. Male predominance less pronounced.	Predominantly affects males, largely due to higher rates of tobacco and alcohol use
Risk factors	Often occurs in nonsmokers and nondrinkers; associated with genetic predisposition (e.g., CDKN2A, EGFR), epigenetic alterations, oral microbiome dysbiosis, high sugar intake, and vaping	Strongly linked to cumulative exposure to tobacco and alcohol; also associated with betel quid use in endemic regions.
Tumor location	Primarily affects the anterior two‐thirds and lateral borders of the tongue.	Common sites include the tongue, floor of mouth, gingiva, and buccal mucosa.
Molecular profile	Lower tumor mutational burden; frequent mutations in TP53, CDKN2A, CASP8, NOTCH1, FAT1, and EGFR; activation of Wnt/β‐catenin and NOTCH pathways; early DNA methylation changes.	Higher mutational burden due to carcinogen exposure (tobacco, alcohol); frequent TP53 and CDKN2A mutations; prominent DNA damage signatures.
HPV status	Predominantly HPV‐negative, suggesting potentially distinct oncogenic mechanisms compared with HPV‐driven cancers.	Rarely HPV‐positive; HPV‐driven carcinomas predominantly occur in the oropharynx rather than the oral cavity.
Prognosis	“Prognostic paradox”: despite aggressive histopathology and higher recurrence rates, overall survival is comparable or superior due to better treatment tolerance and fewer comorbidities.	Prognosis depends largely on tumor stage and comorbidities; generally associated with lower survival and increased risk of distant metastases.
Survival outcomes	Comparable or slightly improved overall survival; however, lower disease‐free survival due to earlier recurrence.	Lower overall survival, often due to late‐stage diagnosis and limited treatment tolerance in older patients.

Abbreviations: CASP8 = Caspase 8; CDKN2A = Cyclin‐Dependent Kinase Inhibitor 2A; EGFR = Epidermal Growth Factor Receptor; EO‐OSCC = Early‐Onset Oral Squamous Cell Carcinoma; FAT1 = FAT Atypical Cadherin 1; HPV = Human Papillomavirus; NOTCH1 = Notch receptor 1; OSCC = Oral Squamous Cell Carcinoma; TP53 = Tumor Protein p53; Wnt/β‐catenin = Wingless‐related integration site/β‐catenin.

## 5 Current and Emerging Risk Factors in EO‐OSCC

The etiopathogenesis of EO‐OSCC is multifactorial and remains incompletely understood. Beyond traditional carcinogens, increasing evidence implicates viral and microbial agents, dietary and metabolic factors, chronic inflammation, and behavioral determinants, which may converge on shared pathways of epithelial dysregulation, immune modulation, and genomic instability [3, 5, 6, 13].

**FIGURE 2:**
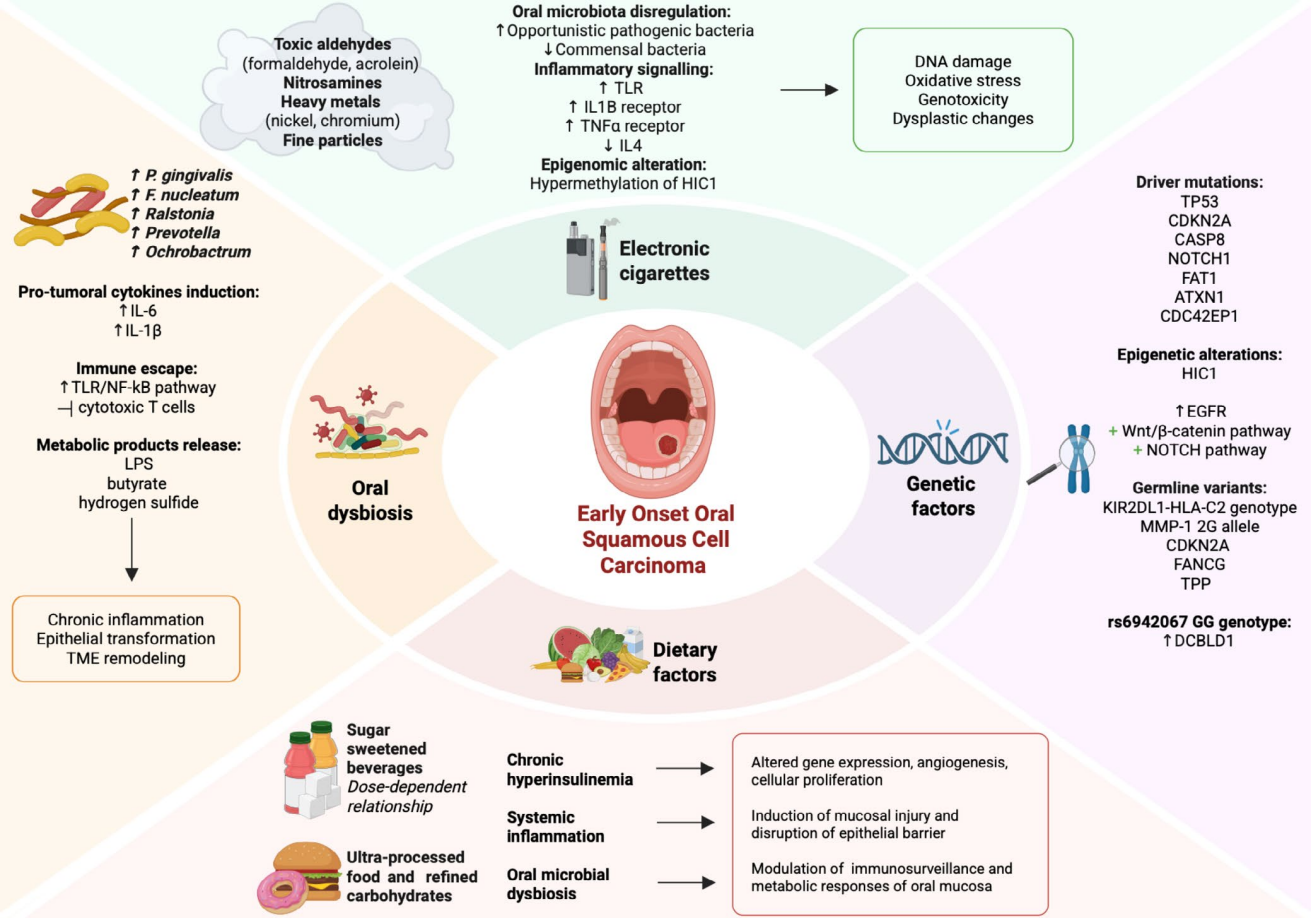
**Overview of major risk factors and proposed carcinogenic pathways involved in EO‐OSCC.** This schematic figure summarizes emerging environmental, biological, and genetic factors that have been proposed to contribute to the pathogenesis of EO‐OSCC, based on heterogeneous epidemiological, molecular, and experimental studies. The depicted mechanisms should be interpreted as hypothesis‐generating rather than established causal pathways. **Oral dysbiosis (left side of the panel, yellow):** Age‐associated alterations in the oral microbiome have been reported in EO‐OSCC, including increased abundance of *P. gingivalis* , *Ralstonia*, *Prevotella*, and *Ochrobactrum*, while *F. nucleatum* remains abundant across age groups. Microbial dysbiosis may promote pro‐tumoral processes shared with conventional OSCC, such as induction of inflammatory cytokines (IL‐6, IL‐1β), activation of TLR/NF‐κB signaling, suppression of cytotoxic T‐cell activity, and release of carcinogenic microbial metabolites (e.g., lipopolysaccharide, butyrate, hydrogen sulfide). These mechanisms may contribute to chronic inflammation, epithelial transformation, and remodeling of the tumor microenvironment. **Electronic cigarette exposure (top of the panel, green):** Components of electronic cigarette aerosols, including toxic aldehydes (formaldehyde, acrolein), nitrosamines, heavy metals, and fine particles may disrupt oral microbial homeostasis and enhance colonization by opportunistic and pathogenic species. Associated alterations in inflammatory signaling pathways (e.g., TLR, IL‐1β receptor, TNF‐α receptor, IL‐4) and epigenetic changes, such as HIC1 hypermethylation, have been proposed to promote oxidative stress, DNA damage, genotoxicity, and dysplastic epithelial changes. **Genetic factors (right side of the panel, pink):** EO‐OSCC has been associated with a molecular profile that differs from that of conventional OSCC in several studies. Recurrent driver mutations have been reported, including TP53, CDKN2A, CASP8, NOTCH1, FAT1, ATXN1, and CDC42EP1, with TP53 and CDKN2A being most frequently involved. These alterations may coexist with epigenetic dysregulation, such as promoter hypermethylation of HIC1, amplification of EGFR, and activation of developmental pathways including Wnt/β‐catenin and NOTCH signaling. In addition, germline variants (e.g., KIR2DL1–HLA‐C2, MMP‐1 2G allele, CDKN2A, FANCG, TPP) and the rs6942067 GG genotype, associated with increased DCBLD1 expression, suggest a heritable susceptibility component in a subset of patients. **Dietary factors (bottom of the panel, orange):** Emerging epidemiologic evidence suggests that high intake of sugar‐sweetened beverages, ultra‐processed foods, and refined carbohydrates may be associated with an increased risk of EO‐OSCC in a dose‐dependent manner. Proposed mechanisms include chronic hyperinsulinemia, systemic inflammation, and diet‐induced microbial dysbiosis that can modulate gene expression, angiogenesis, cellular proliferation, and immune regulation within the oral mucosa. Abbreviations: ATXN1 = Ataxin‐1; CASP8 = Caspase‐8; CDKN2A = Cyclin‐Dependent Kinase Inhibitor 2A; CDC42EP1 = CDC42 Effector Protein 1; DCBLD1 = Discoidin, CUB and LCCL Domain‐Containing Protein 1; EGFR = Epidermal Growth Factor Receptor; EO‐OSCC = Early‐Onset Oral Squamous Cell Carcinoma; FAT1 = FAT Atypical Cadherin 1; FANCG = Fanconi Anemia Complementation Group G; F. nucleatum = Fusobacterium nucleatum; HIC1 = Hypermethylated in Cancer 1; IL‐1β = Interleukin‐1 beta; IL‐4 = Interleukin‐4; IL‐6 = Interleukin‐6; KIR2DL1‐HLA‐C2 = Killer Cell Immunoglobulin‐like Receptor 2DL1 Human‐Leukocyte Antigen C2 group; LPS = Lipopolysaccharide; MMP‐1 = Matrix Metalloproteinase‐1; NOTCH1 = Notch Receptor 1; OSCC = Oral Squamous Cell Carcinoma; P.gingivalis = Porphyromonas gingivalis; SSB = Sugar‐Sweetened Beverages; TME = Tumor Microenvironment; TLR/NF‐κB = Toll‐Like Receptor/Nuclear Factor kappa‐light‐chain‐enhancer of activated B cells; TNF‐α = Tumor Necrosis Factor α; TP53 = Tumor Protein p53; TPP = Tubulin Polymerization‐Promoting Protein; Wnt/β catenin = Wingless‐related integration site/β‐catenin.

Figure 2 summarizes the principal determinants and mechanistic hypotheses currently proposed in EO‐OSCC.

### 5.1 Traditional Exposures: Tobacco, Alcohol, and Shifting Patterns in Young Patients

Tobacco and alcohol remain established risk factors in EO‐OSCC, although their prevalence is consistently lower than in older patients. A meta‐analysis reported tobacco use in 39.5% of younger individuals versus 48.4% in older cohorts, and alcohol consumption in 30.9% versus 45.8%, respectively [3]. These findings indicate a reduced but still relevant contribution of traditional carcinogens in early‐onset disease.

### 5.2 Electronic Cigarette Use and Vaping: Emerging Carcinogenic Concerns

The increasing use of electronic cigarettes (e‐cigarettes) among adolescents and young adults has raised concerns regarding their potential role in oral carcinogenesis. Vaping aerosols contain aldehydes, volatile organic compounds, and heavy metals capable of inducing DNA damage, oral microbiota dysregulation, oxidative stress, inflammatory signaling (marked by reduced interleukin (IL)‐4 and increased Toll‐like receptor (TLR) signaling, with upregulation of proinflammatory cytokines, including Tumor necrosis factor‐α (TNF‐α) and IL‐1β), and epithelial dysplasia in experimental models [14]. Emerging evidence also suggests that vaping may promote epigenetic dysregulation, including hypermethylation and functional silencing of tumor suppressor genes, such as Hypermethylated in Cancer 1 (HIC1), providing a plausible mechanistic link to early carcinogenic events [15].

Although epidemiologic data directly linking vaping to EO‐OSCC remain limited, its high prevalence in younger populations supports its consideration as a plausible modifiable risk factor. Accordingly, nicotine‐containing e‐cigarettes have been classified as potentially carcinogenic by the Clinical Oncology Society of Australia [16]. Overall, current evidence supports a hypothesis‐generating role for e‐cigarette exposure in EO‐OSCC, warranting further investigation through longitudinal and population‐based studies.

### 5.3 HPV: Site‐Specific Relevance and Ongoing Controversies

The etiologic role of HPV in EO‐OSCC remains controversial and appears strongly site‐dependent.

While HPV is a well‐established driver of oropharyngeal squamous cell carcinoma, its contribution to cancers of the oral cavity is considerably less consistent. Although early studies suggested a possible association in nonsmokers and nondrinkers (NSND), more recent evidence indicates low HPV positivity rates in EO‐OSCC, particularly in lateral tongue tumors, arguing against a dominant causal role [17, 18]. Overall, available evidence suggests that HPV accounts for only a limited subset of EO‐OSCC cases and does not adequately explain the rising incidence observed in young adults. Future studies should employ standardized HPV detection methods and report results by specific oral subsites to better clarify its true etiologic relevance in EO‐OSCC.

### 5.4 Betel Quid, Areca Nut, and Regional Risk Factors

Betel quid and areca nut chewing remain major carcinogenic exposures in South and Southeast Asia and are classified as Group 1 carcinogens by the International Agency for Research on Cancer [19, 20]. Mechanistically, areca nut–derived alkaloids, including arecoline, have been shown to induce genotoxic stress, epigenetic alterations, and chronic mucosal inflammation, creating a microenvironment conducive to malignant transformation. However, many EO‐OSCC cases in Western populations occur without such exposure, indicating that betel quid represents a region‐specific rather than universal risk factor and supporting geographically tailored prevention strategies [19, 20].

### 5.5 Dietary Exposures and Nutritional Patterns in EO‐OSCC

Dietary and metabolic factors are increasingly recognized as potential contributors to EO‐OSCC, particularly among NSND. A large prospective cohort study involving over 160 000 women aged 27–44 years reported a strong dose–response association between sugar‐sweetened beverage (SSB) intake and oral cavity cancer risk (HR 4.87; 95% CI, 2.47–9.60), which increased to 5.46 (95% CI, 1.75–17.07) among NSND [21]. Proposed mechanisms linking high sugar intake to oral carcinogenesis include chronic hyperinsulinemia, systemic inflammation, and diet‐induced alterations of the oral microbiome, which may influence epithelial proliferation, angiogenesis, and immune regulation [22, 23].

Beyond SSBs, overall dietary quality may modulate OSCC risk. Diets rich in fruits, vegetables, and polyphenols, as well as consumption of green tea and coffee, both sources of bioactive compounds such as epigallocatechin‐3‐gallate, have shown anticancer effects via antioxidant and anti‐inflammatory mechanisms [24].

Broader dietary patterns emphasizing plant‐based foods and limiting processed meats and refined carbohydrates have also been linked to OSCC risk modification [25].

Although current evidence remains largely observational, these findings support a plausible role for modifiable dietary exposures in EO‐OSCC and highlight the need for prospective studies using validated nutritional biomarkers.

### 5.6 Oral Hygiene, Microbiome Dysbiosis, and Inflammation‐Driven Carcinogenesis

Growing evidence supports a role for oral microbiome dysbiosis in carcinogenesis through epithelial disruption, genotoxic metabolite production, immune modulation, and chronic inflammation [23, 26]. *Fusobacterium spp.* and *Porphyromonas gingivalis* are consistently implicated in pro‐inflammatory signaling and tumor‐promoting microenvironmental changes, with the induction of pro‐tumoral cytokines (e.g., IL‐6, IL‐1β), and immune escape via cytotoxic T cell suppression, TLR modulation and Nuclear Factor kappa‐light‐chain‐enhancer of activated B cells (NF‐kB) activation [26].

Metatranscriptomic analyses in nonsmoking individuals have demonstrated increased *Fusobacteria* activity in tumor and peritumoral tissues compared with healthy controls, suggesting a functional contribution to carcinogenesis [27]. Emerging data further indicate that EO‐OSCC may exhibit age‐related microbiome alterations, with enrichment of genera such as *Ralstonia*, *Prevotella*, and *Ochrobactrum*, while *Fusobacterium* remains prevalent across age groups [23].

In this context, oral hygiene represents a clinically relevant and potentially modifiable factor. Poor oral hygiene and periodontal disease may sustain microbial dysbiosis and chronic local inflammation, thereby interacting with other exposures such as diet, tobacco, and vaping to promote inflammation‐driven carcinogenic pathways. Although EO‐OSCC–specific data remain limited, incorporating oral hygiene and microbiome‐related variables into EO‐OSCC risk models appears biologically plausible and warrants further investigation [28].

## 6 Molecular Mechanisms

EO‐OSCC exhibits molecular features distinct from conventional OSCC, supporting a differentiated tumor biology [4, 29]. Genomic analyses on 227 oral tongue squamous cell carcinoma samples including 107 EO‐OSCC cases identified seven putative driver genes: Tumor Protein p53 (TP53), Cyclin‐Dependent Kinase Inhibitor 2A (CDKN2A), Caspase 8 (CASP8), Notch Receptor 1 (NOTCH1), FAT Atypical Cadherin 1 (FAT1), Ataxin 1 (ATXN1), and CDC42 Effector Protein 1 (CDC42EP1), with TP53 and CDKN2A being the most frequently involved [13]. Notably, EO‐OSCC tumors also display a lower somatic mutational burden compared with typical‐onset OSCC, not fully explained by tobacco exposure. These genetic alterations are often accompanied by epigenetic dysregulation, including promoter hypermethylation and silencing of genes involved in DNA repair and cell‐cycle regulation [4]. Transcriptomic and epigenomic analysis further suggest altered chromatin remodeling and activation of developmental pathways such as Wnt/β‐catenin and NOTCH signaling, supporting proliferative and invasive phenotypes. Distinct gene expression and noncoding RNA profiles have also been reported compared with older patients, although these observations derive from limited sample sizes and should be interpreted with caution [4, 30]. Moreover, molecular subgroups characterized by Epidermal Growth Factor Receptor (EGFR) amplification and downstream pathway activation have been described, highlighting potential targets for biomarker‐driven therapies [5, 31]. Emerging evidence also supports a contribution of inherited susceptibility, including variants involving Killer Cell Immunoglobulin‐like Receptor 2DL1–Human Leukocyte Antigen C2 (KIR2DL1–HLA‐C2) genotype and the Matrix Metalloproteinase‐1 (MMP)‐1 2G allele [29, 30]. Although metabolic reprogramming and chronic inflammation are features shared with conventional OSCC, in EO‐OSCC these processes may be influenced by host‐specific factors such as inherited cancer syndromes, immune dysregulation, or other germline susceptibilities [4, 29].

Consistent with the conceptual framework summarized in Figure 2, EO‐OSCC carcinogenesis likely reflects the interaction between genetic predisposition and modifiable exposures, including oral dysbiosis, dietary patterns, and emerging factors such as electronic cigarette use.

## 7 Immune Microenvironment

The tumor microenvironment (TME) of EO‐OSCC differs from that of conventional OSCC in both cellular composition and immune regulation, with most studies supporting a predominantly immunosuppressive phenotype [6]. Hallmark features include enhanced epithelial‐to‐mesenchymal transition, extracellular matrix remodeling, increased oxidative stress, and elevated MMP‐9 expression, collectively promoting invasion and metastatic potential [30]. Immunologically, EO‐OSCC exhibits reduced infiltration of CD8^+^ cytotoxic T lymphocytes together with enrichment of immunosuppressive populations such as regulatory T cells and myeloid‐derived suppressor cells (MDSCs), accompanied by increased expression of immune checkpoint molecules, including programmed death‐ligand 1 (PD‐L1) and cytotoxic T‐lymphocyte–associated protein 4 (CTLA‐4) [6]. Spatial heterogeneity is frequently observed, with immune cells concentrated at the invasive front and relative immune exclusion within the tumor core, a pattern associated with reduced responsiveness to immunotherapy [30].

Host genetic background further shapes immune landscape. Germline variants involving antigen presentation and immune regulation, including MHC class I polypeptide‐related sequence A (MICA) A5.1 homozygosity and alterations in CDKN2A, Fanconi Anemia Complementation Group G (FANCG), and Tubulin Polymerization‐Promoting Protein (TPP), have been associated with reduced immune infiltration. In addition, the rs6942067 GG genotype has been linked to upregulation of Discoidin, CUB and LCCL Domain‐Containing Protein 1 (DCBLD1) and immune resistance, particularly in HPV‐negative NSND EO‐OSCC [32, 33]. Among emerging biomarkers, galectin‐7 has been correlated with aggressive behavior and resistance to chemoradiotherapy in younger patients [6]. Additional biomarkers, such as the chromatin modifier Enhancer of Zeste Homolog 2 (EZH2), Excision Repair Cross‐Complementation Group 1 (ERCC1), estrogen receptor (ER), and copy number variations (CNVs), may also contribute to therapeutic stratification, as their dysregulation has been implicated in tumor aggressiveness, DNA repair capacity, and hormone‐related signaling pathways [30, 34, 35]. Overall, these features support an immune‐excluded (“cold”) tumor phenotype.

**FIGURE 3:**
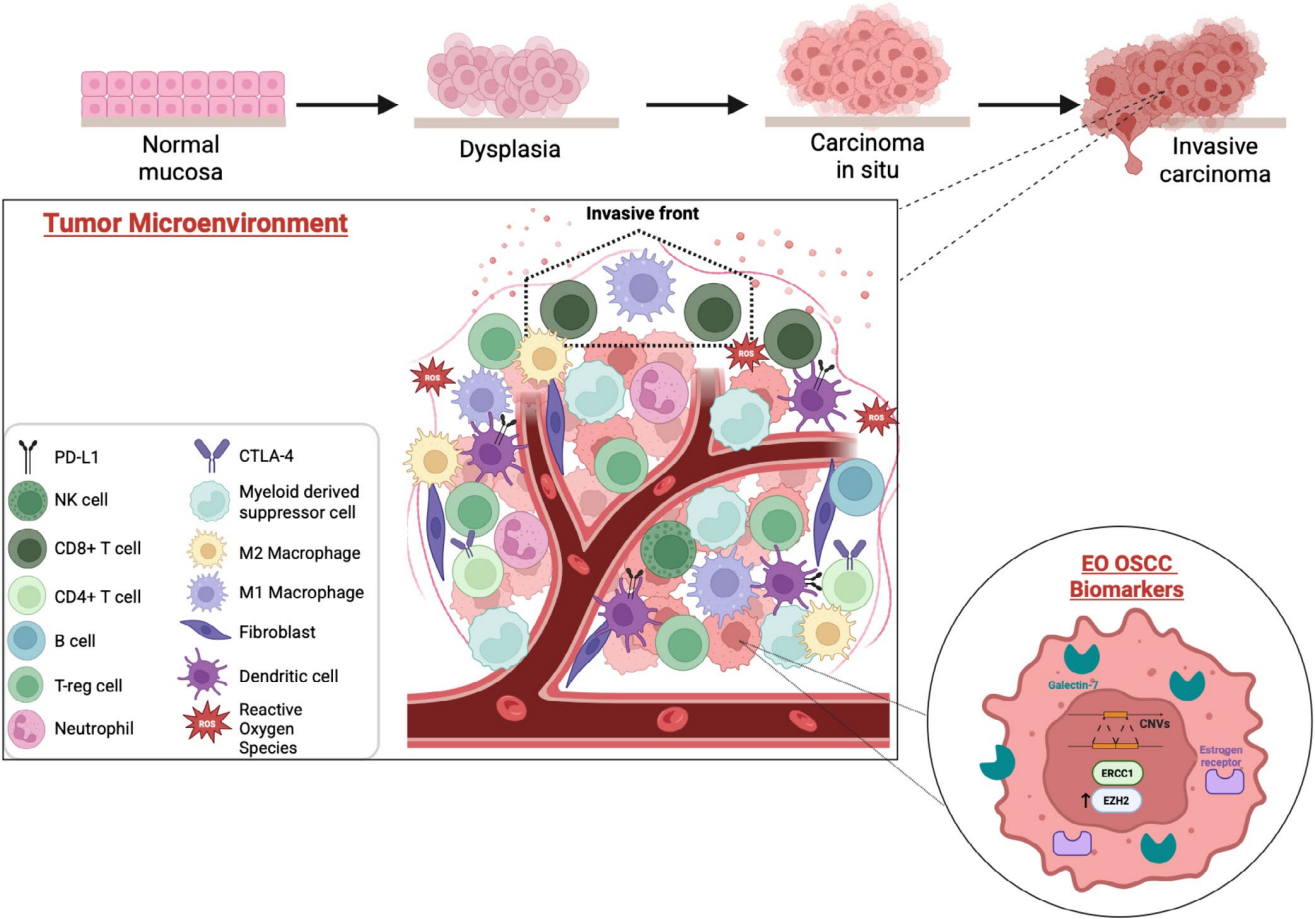
**Schematic representation of the TME in EO‐OSCC.** The **upper panel** illustrates the histologic continuum of oral carcinogenesis, progressing from normal mucosa through epithelial dysplasia and carcinoma in situ, to invasive carcinoma. The **figure** **at** **the** **center** depicts key components of the TME in EO‐OSCC, which in several studies differs from that observed in conventional OSCC. EO‐OSCC is characterized by decreased infiltration of CD8^+^ T cells, and increased presence of immunosuppressive T‐reg cells and MDSCs. At the invasive front, CD8^+^ T cells and M1 macrophages are more frequently observed, whereas the tumor core often exhibits an immune‐excluded phenotype. This spatial organization has been associated with reduced responsiveness to immune checkpoint inhibitors and primary resistance to immunotherapy. Increased expression of immune checkpoint molecules, including PD‐L1 and CTLA‐4, further contributes to local immune suppression. Additional features include enhanced EMT, ECM remodeling, increased ROS levels, and increased MMP‐9 expression, supporting tumor invasion and metastatic potential. The **magnified inset (bottom right)** highlights emerging biomarkers reported in EO‐OSCC, including differential expression of galectin‐7, ERCC1, EZH2, estrogen receptor signaling, and distinct CNVs, which have been associated with immunosuppressive phenotypes and clinical outcomes. The clinical and prognostic implications of these biomarkers are summarized in Table 3. Abbreviations: CD8+ cell = Cluster of Differentiation 8 positive cell; CD4+ cell = Cluster of DIfferentiation 4 positive cell; CNVs = Copy Number Variations; CTLA‐4 = Cytotoxic T‐Lymphocyte–Associated Protein 4; ECM = Extracellular Matrix; EMT = Epithelial‐to‐Mesenchymal Transition; EO‐OSCC = Early‐Onset Oral Squamous Cell Carcinoma; ERCC1 = Excision Repair Cross‐Complementation group 1; EZH2 = Enhancer of Zeste Homolog 2; M1 Macrophage = Classically activated Macrophage; M2 Macrophage = Alternatively activated macrophage; MDSCs = Myeloid‐Derived Suppressor Cells; MMP‐9 = Matrix Metalloproteinase‐9; NK cell = Natural Killer cell; NLR = Neutrophil‐to‐Lymphocyte Ratio; OSCC = Oral Squamous Cell Carcinoma; PD‐L1 = Programmed Death‐Ligand 1; ROS = Reactive Oxygen Species; TME = Tumor Microenvironment; T‐reg = regulatory T cells.

Table 3 summarizes the principal molecular and immunological biomarkers investigated in EO‐OSCC, highlighting their biological roles, expression patterns, and potential clinical implications, and Figure 3 provides an overview of the EO‐OSCC immune microenvironment.

**TABLE 3:** Molecular and immune biomarkers associated with EO‐OSCC.

Biomarker	Molecular function/pathway	Expression pattern in EO‐OSCC	Clinical/prognostic implication
TP53 [4]	Tumor suppressor, cell‐cycle regulation	Mutation/loss common	Associated with poor differentiation
CDKN2A/p16 [30]	Cell‐cycle checkpoint	Promoter methylation, inactivation	Distinguishes EO‐OSCC from HPV+ tumors
EGFR [5]	Growth factor receptor	Amplification, pathway activation	Target for EGFR inhibitors (e.g., Afatinib)
EZH2 [30]	Epigenetic regulator (PRC2 complex)	Overexpression	Correlates with aggressiveness, poor prognosis
Galectin‐7 [6]	Apoptosis and adhesion modulator	Upregulated in EO‐OSCC	Marker of chemoradioresistance
PD‐L1 [6]	Immune checkpoint	Overexpressed	Indicates immunosuppressive microenvironment

Abbreviations: CDKN2A/p16 = Cyclin‐Dependent Kinase Inhibitor 2A/p16INK4a; EGFR = Epidermal Growth Factor Receptor; EO‐OSCC = Early‐Onset Oral Squamous Cell Carcinoma; EZH2 = Enhancer of Zeste Homolog 2; HPV = Human Papillomavirus; PD‐L1 = Programmed Death‐Ligand 1; PRC2 = Polycomb Repressive Complex 2; TP53 = Tumor Protein p53.

## 8 Clinical Description and Histology

EO‐OSCC in young adults is frequently under‐recognized, with diagnostic delays particularly common among patients lacking classical risk exposures. The anterior two‐thirds of the tongue represent the predominant primary site (≈59.9%–89%; pooled ~72%), while gingiva, buccal mucosa, and lips are less commonly involved [2, 36].

Clinically, EO‐OSCC often presents as persistent painless ulcers, leukoplakia, erythroplakia, or exophytic lesions, underscoring the importance of vigilance during routine oral examinations, especially for lesions persisting beyond 2–3 weeks [8, 10, 37].

Histopathological evaluation remains the diagnostic gold standard. Tumors are most frequently keratinizing squamous cell carcinomas with moderate differentiation, although poorly differentiated forms and aggressive features, such as perineural (38%) and lymphovascular invasion (9%) are commonly observed and associated with adverse outcomes [36]. Prognostic markers, including tumor budding and depth of invasion, further refine risk stratification [38]. Although not specific to EO‐OSCC, these histopathologic markers are likely relevant in younger patients and may improve risk stratification.

Digital pathology and artificial intelligence (AI) have shown expert‐level diagnostic performance in OSCC, suggesting potential utility in EO‐OSCC, particularly in challenging cases and resource‐limited settings [39]. Adjunctive tools such as toluidine blue staining and narrow‐band imaging may support clinical assessment, but definitive diagnosis relies on histopathologic confirmation [13, 36].

## 9 Prognostic Complexity Patterns and Biological Determinants

Early reports described EO‐OSCC as an aggressive disease, with higher rates of poor differentiation, perineural invasion, and locoregional failure, supporting the use of intensified treatment strategies in younger patients [7, 40]. Subsequent evidence has revealed a heterogeneous prognostic pattern. Some studies reported improved outcomes in younger patients, with up to a 50% reduction in mortality risk [8], whereas others observed worse survival compared with older individuals [41]. However, pooled meta‐analyses consistently demonstrated comparable OS between EO‐OSCC and conventional OSCC, indicating that age alone is not a dominant prognostic determinant [2]. More recent population‐based analyses have described a “prognostic paradox,” whereby EO‐OSCC patients, despite aggressive histopathologic features, demonstrate equivalent or improved OS, likely reflecting fewer comorbidities, better treatment tolerance, and superior performance status [42]. Panda et al. reported improved OS in younger adults (OR 1.48; 95% CI 1.09–2.01), while Chen et al. observed significantly higher 5‐year overall and disease‐specific survival [42, 43]. In contrast, disease‐specific outcomes suggest increased biological aggressiveness, with worse disease‐free survival and higher rates of local recurrence and distant metastasis in EO‐OSCC [42, 44]. Emerging evidence links poorer outcomes to unfavorable immune profiles, including immune‐cold phenotypes and reduced tumor‐infiltrating lymphocytes, as well as elevated systemic inflammatory markers such as the neutrophil‐to‐lymphocyte ratio (NLR) [30, 45]. Overall, EO‐OSCC appears to combine relatively preserved overall survival with increased recurrence risk, supporting the need for age‐specific prognostic models integrating clinical, molecular, immunologic, and histopathologic parameters.

## 10 Clinical Management

The management of EO‐OSCC requires balancing oncologic control with long‐term functional preservation. Although treatment principles mirror those of conventional OSCC, younger patients often receive more intensive multimodal approaches [8]. Surgical resection remains the primary treatment modality. Early‐onset patients are more frequently treated with surgery followed by adjuvant radiotherapy, likely reflecting attempts to counteract aggressive tumor features; when matched for stage and nodal status, survival outcomes are comparable across age groups [8, 46]. Chemotherapy regimens do not differ substantially by age; however, in selected cases, neoadjuvant approaches have been explored. Taxane‐based regimens preceding surgery have shown promise in improving local tumor control with acceptable toxicity profiles [47]. However, the potential long‐term impacts, including secondary malignancies, fertility impairment, and neurotoxicity, warrant careful consideration in younger patients [48].

Targeted therapies represent an emerging option in molecularly selected subgroups. EGFR inhibition, including afatinib, has demonstrated activity, particularly in HPV‐negative disease [31]. Radiotherapy protocols remain comparable across age groups, but younger patients often tolerate intensified regimens, potentially improving locoregional control at the cost of increased late toxicities such as xerostomia, osteoradionecrosis, and radiation‐induced second tumors [46, 49]. Moreover, clinical management of EO‐OSCC should account not only for biological risk factors, but also for psychosocial and behavioral determinants that may affect diagnosis, treatment, and survivorship outcomes. Diagnostic delays are common, particularly among NSND, and are attributed to low perceived cancer risk, misinterpretation of early lesions, and delayed referral to specialist care. In addition, younger survivors often experience prolonged functional and psychosocial burden, including eating and speech difficulties, altered body image, anxiety, depression, and social isolation, underscoring the need for integrated supportive care within multidisciplinary management [49, 50].

## 11 Conclusion

EO‐OSCC represents a biologically and clinically distinct subset of oral cancer, often arising in patients without traditional carcinogenic exposures. Despite frequently aggressive histologic patterns, OS is commonly preserved, reflecting a “prognostic paradox” likely driven by host‐related factors, treatment tolerance, and age‐specific tumor–host interactions. Distinct molecular alterations and an immunosuppressive, spatially heterogeneous TME appear to modulate disease behavior and may underlie the dissociation between OS and recurrence risk observed in younger patients.

Integrating molecular profiling, immune contexture, and refined histopathologic parameters holds promise for improved risk stratification and individualized management. Emerging approaches, including AI‐assisted diagnostics and immunogenomic profiling, may further enhance early detection and therapeutic decision‐making, improving the quality of life in younger patients.

## Author Contributions

**Gennaro Musella:** conceptualization; methodology; investigation; writing – original draft. **Cristina D'Antonio:** methodology; investigation; data curation; writing – original draft; visualization. **Federica Canfora:** investigation; data curation; writing – review and editing. **Michele D. Mignogna:** writing – review and editing; supervision. **Valentino Vellone:** visualization; writing – review and editing. **Vito Carlo Alberto Caponio:** validation; writing – review and editing. **Amerigo Giudice:** supervision; writing – review and editing. **Alessandro Villa:** conceptualization; supervision; writing – review and editing. **Daniela Adamo:** conceptualization; supervision; project administration; writing – review and editing.

## Funding

The authors have nothing to report.

## Disclosure

The authors used an AI‐based tool exclusively for English language editing and stylistic refinement. No AI tools were used to generate scientific content, analyze data, interpret results, or draw conclusions. All content was reviewed and approved by the authors, who take full responsibility for the accuracy and integrity of the manuscript.

## Conflicts of Interest

The authors declare no conflicts of interest.

## Supporting information


**Table S1:** Studies included in Figure 1.

## Data Availability

Data sharing is not applicable to this article as no datasets were generated or analysed during the current study.

## Appendix A

**Reference list of articles included in Table 1**

1. Myers JN, Elkins T, Roberts D, Byers RM. Squamous cell carcinoma of the tongue in young adults: Increasing incidence and factors that predict treatment outcomes. *Otolaryngol Neck Surg*. 2000;122(1):44‐51. doi:10.1016/S0194‐5998(00)70142‐2
2. Müller S, Pan Y, Li R, Chi AC. Changing trends in oral squamous cell carcinoma with particular reference to young patients: 1971‐2006. The Emory University experience. *Head Neck Pathol*. 2008;2(2):60‐66. doi:10.1007/s12105‐008‐0054‐5
3. Sherin N, Simi T, Shameena P, Sudha S. Changing trends in oral cancer. *Indian J Cancer*. 2008;45(3):93‐96. doi:10.4103/0019‐509x.44063
4. Patel SC, Carpenter WR, Tyree S, et al. Increasing incidence of oral tongue squamous cell carcinoma in young white women, age 18 to 44 years. *J Clin Oncol Off J Am Soc Clin Oncol*. 2011;29(11):1488‐1494. doi:10.1200/JCO.2010.31.7883
5. McGorray SP, Guo Y, Logan H. Trends in incidence of oral and pharyngeal carcinoma in Florida: 1981‐2008. *J Public Health Dent*. 2012;72(1):68‐74. doi:10.1111/j.1752‐7325.2011.00285.x
6. Tota JE, Anderson WF, Coffey C, et al. Rising incidence of oral tongue cancer among white men and women in the United States, 1973‐2012. *Oral Oncol*. 2017;67:146‐152. doi:10.1016/j.oraloncology.2017.02.019
7. Al‐Jamaei A a. H, van Dijk B a. C, Helder MN, Forouzanfar T, Leemans CR, de Visscher JG a. M. A population‐based study of the epidemiology of oral squamous cell carcinoma in the Netherlands 1989‐2018, with emphasis on young adults. *Int J Oral Maxillofac Surg*. 2022;51(1):18‐26. doi:10.1016/j.ijom.2021.03.006
8. Ferreira E Costa R, Leão MLB, Sant’Ana MSP, et al. Oral Squamous Cell Carcinoma Frequency in Young Patients from Referral Centers Around the World. *Head Neck Pathol*. 2022;16(3):755‐762. doi:10.1007/s12105‐022‐01441‐w
9. Deneuve S, Pérol O, Dantony E, et al. Diverging incidence trends of oral tongue cancer compared to other head and neck cancers in young adults in France. *Int J Cancer*. 2022;150(8):1301‐1309. doi:10.1002/ijc.33896
10. Révész M, Oberna F, Slezák A, Ferenczi Ö, Kenessey I, Takácsi‐Nagy Z. The characteristics of head and neck squamous cell cancer in young adults: A retrospective single‐center study. *Pathol Oncol Res POR*. 2023;29:1611123. doi:10.3389/pore.2023.1611123
11. Sakr Y, Hamdy O, Eldeghedi M, et al. Shifting Epidemiology Trends in Tongue Cancer: A Retrospective Cohort Study. *Cancers*. 2023;15(23):5680. doi:10.3390/cancers15235680
12. da Silva Souto AC, da Silva BNM, Heimlich FV, et al. Epidemiological landscape of tongue cancer in younger patients in a National Cancer Center in Brazil. *Sci Rep*. 2024;14(1):30573. doi:10.1038/s41598‐024‐83470‐9
13. Singh M, Thankappan K, Balasubramanian D, et al. Contrasting clinical outcomes and socio‐economic impact of young versus elderly‐onset oral squamous cell carcinoma, a novel health economic analysis. *Cancer Med*. 2024;13(3):e6747. doi:10.1002/cam4.6747
14. Sufiawati I, Piliang A, Yusuf AA, et al. Clinicopathological Characteristics of Oral Squamous Cell Carcinoma at the Central Referral and Teaching Hospital in West Java, Indonesia. *Cancer Manag Res*. 2024;16:1053‐1061. doi:10.2147/CMAR.S476557
15. Szewczyk M, Pazdrowski J, Golusiński P, Więckowska B, Golusiński W. Oral cancer in young adults: should we approach these patients differently? *Front Oncol*. 2024;14:1297752. doi:10.3389/fonc.2024.1297752
16. Hong X, Quimby AE, Mavedatnia D, et al. Oral Cavity Squamous Cell Carcinoma in Young Patients: A Multi‐Institutional Study of the Canadian Head & Neck Collaborative Research Initiative. *J Otolaryngol—Head Neck Surg J Oto‐Rhino‐Laryngol Chir Cervico‐Faciale*. 2025;54:19160216251351562. doi:10.1177/19160216251351562
