# Supplementary material for: Early‐Onset Oral Squamous Cell Carcinoma: Emerging Biological Insights, Risk Factors and Clinical Implications
Source: J Oral Pathol Med. 2026 Mar 15;55(6):629–41. doi: 10.1111/jop.70130 (PMC13333533; doi:10.1111/jop.70130)
Supplement: Supplementary file 1 — Table S1: Studies included in Figure 1. [file JOP-55-629-s001.docx]

**Table S1. Studies included in Figure 1.**

| **Authors** | **Year** | **Region** | **Country** |
| --- | --- | --- | --- |
| Chang et al. [1] | 2013 | Asia | China |
| Fan et al. [2] | 2014 | Asia | China |
| Ho et al. [3] | 2008 | Asia | China |
| Li et al. [4] | 2020 | Asia | China |
| Sun et al. [5] | 2015 | Asia | China |
| Xu et al. [6] | 2018 | Asia | China |
| Yu et al. [7] | 2022 | Asia | China |
| Zhang et al. [8] | 2017 | Asia | China |
| Abdulla et al. [9] | 2018 | Asia | India |
| Acharya et al. [10] | 2012 | Asia | India |
| Beena et al. [11] | 2016 | Asia | India |
| Chaware et al. [12] | 2022 | Asia | India |
| Devadass et al. [13] | 2020 | Asia | India |
| Iype et al. [14] | 2004 | Asia | India |
| Manuel et al. [15] | 2003 | Asia | India |
| Iype et al. [16] | 2001 | Asia | India |
| Sharma et al. [17] | 2016 | Asia | India |
| Subramaniam et al. [18] | 2020 | Asia | India |
| Subramaniam et al. [19] | 2018 | Asia | India |
| Ur Rahaman et al. [20] | 2014 | Asia | India |
| Baba et al. [21] | 2022 | Asia | Japan |
| Mizuno et al. [22] | 2020 | Asia | Japan |
| Omura et al. [23] | 2023 | Asia | Japan |
| Tomihara et al. [24] | 2009 | Asia | Japan |
| Yip et al. [25] | 2010 | Asia | Japan |
| Park et al. [26] | 2010 | Asia | North Korea |
| Zaman et al. [27] | 2016 | Asia | Pakistan |
| Mahmood et al. [28] | 2018 | Asia | Pakistan |
| Choi et al. [29] | 2019 | Asia | South Korea |
| Jeon et al. [30] | 2017 | Asia | South Korea |
| Siriwardena et al. [31] | 2006 | Asia | Sri Lanka |
| Rautava et al. [32] | 2007 | Europe | Finland |
| Seppälä et al. [33] | 2021 | Europe | Finland |
| Blanchard et al. [34] | 2017 | Europe | France |
| Deneuve et al. [35] | 2022 | Europe | France |
| Mallet et al. [36] | 2009 | Europe | France |
| Troeltzsch et al. [37] | 2014 | Europe | Germany |
| Csurgay et al. [38] | 2021 | Europe | Hungary |
| Garavello et al. [39] | 2007 | Europe | Italy |
| Mascitti et al. [40] | 2020 | Europe | Italy |
| Ansarin et al. [41] | 2021 | Europe | Italy |
| Al-Jamaei et al. [42] | 2022 | Europe | Netherlands |
| van Monsjou et al. [43] | 2013 | Europe | Netherland |
| Szewczyk et al. [44] | 2024 | Europe | Poland |
| Monteiro et al. [45] | 2018 | Europe | Portugal |
| Cariati et al. [46] | 2017 | Europe | Spain |
| Martin-Granizo et al. [47] | 1997 | Europe | Spain |
| Sasaki et al. [48] | 2005 | Europe | UK |
| Hilly et al. [49] | 2013 | Middle East | Israel |
| Popovtzer et al. [50] | 2004 | Middle East | Israel |
| Soudry et al. [51] | 2010 | Middle East | Israel |
| Vered et al. [52] | 2010 | Middle East | Israel |
| Yosefof et al. [53] | 2020 | Middle East | Israel |
| Yosefof et al. [54] | 2024 | Middle East | Israel |
| Halboub et al. [55] | 2012 | Middle East | Yemen |
| Tremblay et al. [56] | 2006 | North America | Canada |
| Bommakanti et al. [57] | 2023 | North America | USA |
| Campbell et al. [58] | 2019 | North America | USA |
| Farhat et al. [59] | 2022 | North America | USA |
| Farquhar et al. [60] | 2018 | North America | USA |
| Friendlander et al. [61] | 1998 | North America | USA |
| Miller et al. [62] | 2019 | North America | USA |
| Mneimneh et al. [63] | 2021 | North America | USA |
| Morris et al. [64] | 2010 | North America | USA |
| Morris et al. [65] | 2010 | North America | USA |
| Mukdad et al. [66] | 2018 | North America | USA |
| Myers et al. [67] | 2000 | North America | USA |
| Newman et al. [68] | 1983 | North America | USA |
| Oliver et al. [69] | 2019 | North America | USA |
| Rodriguez et al. [70] | 2024 | North America | USA |
| Sturgis et al. [71] | 2005 | North America | USA |
| Valero et al. [72] | 2022 | North America | USA |
| Satgunaseelan et al. [73] | 2021 | Oceania | Australia |
| Satgunaseelan et al. [74] | 2022 | Oceania | Australia |
| Amaral et al. [75] | 2020 | South America | Brazil |
| Barnabé et al. [76] | 2019 | South America | Brazil |
| Borba Ribeiro et al. [77] | 2019 | South America | Brazil |
| Cury et al. [78] | 2021 | South America | Brazil |
| de Matos Amorim et al. [79] | 2023 | South America | Brazil |
| Frare et al. [80] | 2016 | South America | Brazil |
| Hirota et al. [81] | 2008 | South America | Brazil |
| Kaminagakura et al. [82] | 2010 | South America | Brazil |
| Ribeiro et al. [83] | 2009 | South America | Brazil |
| Santos et al. [84] | 2016 | South America | Brazil |
| Tomo et al. [85] | 2020 | South America | Brazil |

**References**

1. Chang TS, Chang CM, Ho HC, et al. Impact of Young Age on the Prognosis for Oral Cancer: A Population-Based Study in Taiwan. Hoque MO, ed. *PLoS ONE*. 2013;8(9):e75855. doi:10.1371/journal.pone.0075855

2. Fan Y, Zheng L, Mao MH, et al. Survival Analysis of Oral Squamous Cell Carcinoma in a Subgroup of Young Patients. *Asian Pac J Cancer Prev*. 2014;15(20):8887-8891. doi:10.7314/APJCP.2014.15.20.8887

3. Ho HC, Lee MS, Hsiao SH, et al. Squamous cell carcinoma of the oral cavity in young patients: a matched-pair analysis. *Eur Arch Otorhinolaryngol*. 2008;265(S1):57-61. doi:10.1007/s00405-007-0496-5

4. Li Y, Hu C. Impact of Age Stratification on the Clinicopathological Characteristics and Survival Outcomes on Stage IV Oral Tongue Squamous Cell Carcinomas. *Cancer Invest*. 2020;38(10):565-571. doi:10.1080/07357907.2020.1830287

5. Sun Q, Fang Q, Guo S. A comparison of oral squamous cell carcinoma between young and old patients in a single medical center in China. *Int J Clin Exp Med*. 2015;8(8):12418-12423.

6. Xu Q, Wang C, Li B, et al. The impact of age on oral squamous cell carcinoma: A longitudinal cohort study of 2,782 patients. *Oral Dis*. 2019;25(3):730-741. doi:10.1111/odi.13015

7. Yu C, Zhou Z. Relationship between young non-smokers and oral cancer: What can we learn? *Oral Oncol*. 2022;133:106064. doi:10.1016/j.oraloncology.2022.106064

8. Zhang YY, Wang DC, Su JZ, Jia LF, Peng X, Yu GY. Clinicopathological characteristics and outcomes of squamous cell carcinoma of the tongue in different age groups. *Head Neck*. 2017;39(11):2276-2282. doi:10.1002/hed.24898

9. Abdulla R, Adyanthaya S, Kini P, Mohanty V, D’Souza N, Subbannayya Y. Clinicopathological analysis of oral squamous cell carcinoma among the younger age group in coastal Karnataka, India: A retrospective study. *J Oral Maxillofac Pathol*. 2018;22(2):180-187. doi:10.4103/jomfp.JOMFP_16_18

10. Acharya S, Tayaar AS. Analysis of clinical and histopathological profiles of oral squamous cell carcinoma in young Indian adults: A retrospective study. *J Dent Sci*. 2012;7(3):224-230. doi:10.1016/j.jds.2012.05.005

11. Beena VT, Binisree SS, Ayswarya T, Paikkadan I, Padmakumar SK, Sivakumar R. Oral Squamous Cell Carcinoma in Patients Younger than 40 Years: A 10 Year Retrospective Study. Int J Sci Stud 2016;4(4):150-153. doi:10.17354/ijss/2016/395

12. Chaware SJ, Sangle VA, Mahajan AM, Gajdhar SS, Sarode SA, Kendre MS. Analysis of Clinicopathological Parameters of Oral Squamous Cell Carcinoma in Different Age Groups. *J Int Clin Dent Res Organ*. 2022;14(2):120-125. doi:10.4103/jicdro.jicdro_38_21

13. Devadass CW, Fathima S, Bhat G, et al. Comparison of Clinicopathological Profile of Oral Squamous Cell Carcinoma between Younger and Older Indian Adults. *J Clin Diagn Res*. Published online 2020. doi:10.7860/JCDR/2020/45660.14241

14. Mathew Iype E, Pandey M, Mathew A, Thomas G, Krishnan Nair M. Squamous cell cancer of the buccal mucosa in young adults. *Br J Oral Maxillofac Surg*. 2004;42(3):185-189. doi:10.1016/j.bjoms.2004.02.008

15. Manuel S, Raghavan SKN, Pandey M, Sebastian P. Survival in patients under 45 years with squamous cell carcinoma of the oral tongue. *Int J Oral Maxillofac Surg*. 2003;32(2):167-173. doi:10.1054/ijom.2002.0271

16. Mathew Iype E, Pandey M, Mathew A, Thomas G, Sebastian P, Krishnan Nair M. Squamous cell carcinoma of the tongue among young Indian adults. *Neoplasia N Y N*. 2001;3(4):273-277. doi:10.1038/sj.neo.7900172

17. Sharma D, Singh G. Squamous cell carcinoma of the oral cavity and oropharynx in young adults. *Indian J Cancer*. 2016;53(3):399. doi:10.4103/0019-509X.200681

18. Subramaniam N, Balasubramanian D, Low THH, et al. Squamous Cell Carcinoma of the Oral Tongue in Young Patients: Outcomes and Implications for Treatment. *Indian J Surg Oncol*. 2020;11(2):274-280. doi:10.1007/s13193-020-01049-y

19. Subramaniam N, Balasubramanian D, Murthy S, Vidhyadharan S, Thankappan K, Iyer S. Oral cancer in the young with no tobacco exposure: A distinct epidemiological subset? *J Head Neck Physicians Surg*. 2018;6(2):86. doi:10.4103/2347-8128.208524

20. Ur Rahaman S, Ahmed Mujib B. Histopathological correlation of oral squamous cell carcinoma among younger and older patients. *J Oral Maxillofac Pathol*. 2014;18(2):183. doi:10.4103/0973-029X.140734

21. Baba D, Matsuura K, Wakabayashi M, et al. Comparison between three age-stratified cohorts reveals poor prognosis of young patients with tongue carcinoma. *Front Oncol*. 2022;12:959749. doi:10.3389/fonc.2022.959749

22. Mizuno K, Takeuchi M, Kikuchi M, Omori K, Kawakami K. Outcomes in patients diagnosed with tongue cancer before and after the age of 45 years. *Oral Oncol*. 2020;110:105010. doi:10.1016/j.oraloncology.2020.105010

23. Omura G, Yoshimoto S, Rikitake R, Eguchi K, Nakamizo M, Nibu KI. Comparison of survival outcomes between adolescent and young adults and older adults with tongue squamous cell carcinoma: a nationwide database study using the head and neck cancer registry of Japan. *Int J Clin Oncol*. 2023;28(2):221-228. doi:10.1007/s10147-022-02279-6

24. Tomihara K, Dehari H, Yamaguchi A, et al. Squamous cell carcinoma of the buccal mucosa in a young adult with history of allogeneic bone marrow transplantation for childhood acute leukemia. *Head Neck*. 2009;31(4):565-568. doi:10.1002/hed.20931

25. Yip CSP, Charn TC, Wee JTS, et al. Outcomes of oral tongue cancer: does age matter? *Ann Acad Med Singapore*. 2010;39(12):897-897.

26. Park JO, Sun DI, Cho KJ, Joo YH, Yoo HJ, Kim MS. Clinical Outcome of Squamous Cell Carcinoma of the Tongue in Young Patients: A Stage-Matched Comparative Analysis. *Clin Exp Otorhinolaryngol*. 2010;3(3):161. doi:10.3342/ceo.2010.3.3.161

27. Shakeel Uz Zaman, ., Adeel, M., Suhail, A. (2016). Squamous cell carcinoma of oral tongue in young patients — A 10 years tertiary care experience.JPMA: Journal of Pakistan Medical Association, 66(2), 155-158.

28. Mahmood N, Hanif M, Ahmed A, Jamal Q, Saqib null, Khan A. Impact of age at diagnosis on clinicopathological outcomes of oral squamous cell carcinoma patients. *Pak J Med Sci*. 2018;34(3):595-599. doi:10.12669/pjms.343.14086

29. Choi G, Song JS, Choi SH, et al. Comparison of Squamous Cell Carcinoma of the Tongue between Young and Old Patients. *J Pathol Transl Med*. 2019;53(6):369-377. doi:10.4132/jptm.2019.09.16

30. Jeon JH, Kim MG, Park JY, et al. Analysis of the outcome of young age tongue squamous cell carcinoma. *Maxillofac Plast Reconstr Surg*. 2017;39(1):41. doi:10.1186/s40902-017-0139-8

31. Siriwardena BSMS, Tilakaratne A, Amaratunga E a. PD, Tilakaratne WM. Demographic, aetiological and survival differences of oral squamous cell carcinoma in the young and the old in Sri Lanka. *Oral Oncol*. 2006;42(8):831-836. doi:10.1016/j.oraloncology.2005.12.001

32. Rautava J, Luukkaa M, Heikinheimo K, Alin J, Grenman R, Happonen RP. Squamous cell carcinomas arising from different types of oral epithelia differ in their tumor and patient characteristics and survival. *Oral Oncol*. 2007;43(9):911-919. doi:10.1016/j.oraloncology.2006.11.012

33. Seppälä M, Jauhiainen L, Tervo S, et al. The expression and prognostic relevance of CDH3 in tongue squamous cell carcinoma. *APMIS*. 2021;129(12):717-728. doi:10.1111/apm.13176

34. Blanchard P, Belkhir F, Temam S, et al. Outcomes and prognostic factors for squamous cell carcinoma of the oral tongue in young adults: a single-institution case-matched analysis. *Eur Arch Oto-Rhino-Laryngol Off J Eur Fed Oto-Rhino-Laryngol Soc EUFOS Affil Ger Soc Oto-Rhino-Laryngol - Head Neck Surg*. 2017;274(3):1683-1690. doi:10.1007/s00405-016-4419-1

35. Deneuve S, Pérol O, Dantony E, et al. Diverging incidence trends of oral tongue cancer compared to other head and neck cancers in young adults in France. *Int J Cancer*. 2022;150(8):1301-1309. doi:10.1002/ijc.33896

36. Mallet Y, Avalos N, Le Ridant AM, et al. Head and neck cancer in young people: a series of 52 SCCs of the oral tongue in patients aged 35 years or less. *Acta Otolaryngol (Stockh)*. 2009;129(12):1503-1508. doi:10.3109/00016480902798343

37. Troeltzsch M, Knösel T, Eichinger C, et al. Clinicopathologic features of oral squamous cell carcinoma: do they vary in different age groups? *J Oral Maxillofac Surg Off J Am Assoc Oral Maxillofac Surg*. 2014;72(7):1291-1300. doi:10.1016/j.joms.2014.01.009

38. Csurgay K, Zalatnai A, Benczik M, et al. A Study of Prognostic Factors in Young Patients With Non-HPV Oral Cancer in Central Europe. *Pathol Oncol Res*. 2021;27:1609991. doi:10.3389/pore.2021.1609991

39. Garavello W, Spreafico R, Gaini RM. Oral tongue cancer in young patients: a matched analysis. *Oral Oncol*. 2007;43(9):894-897. doi:10.1016/j.oraloncology.2006.10.013

40. Mascitti M, Tempesta A, Togni L, et al. Histological features and survival in young patients with HPV‐negative oral squamous cell carcinoma. *Oral Dis*. 2020;26(8):1640-1648. doi:10.1111/odi.13479

41. Ansarin M, De Berardinis R, Corso F, et al. Survival Outcomes in Oral Tongue Cancer: A Mono-Institutional Experience Focusing on Age. *Front Oncol*. 2021;11:616653. doi:10.3389/fonc.2021.616653

42. Al-Jamaei AAH, Van Dijk BAC, Helder MN, Forouzanfar T, Leemans CR, De Visscher JGAM. A population-based study of the epidemiology of oral squamous cell carcinoma in the Netherlands 1989–2018, with emphasis on young adults. *Int J Oral Maxillofac Surg*. 2022;51(1):18-26. doi:10.1016/j.ijom.2021.03.006

43. van Monsjou HS, Wreesmann VB, van den Brekel MWM, Balm AJM. Head and neck squamous cell carcinoma in young patients. *Oral Oncol*. 2013;49(12):1097-1102. doi:10.1016/j.oraloncology.2013.09.001

44. Szewczyk M, Pazdrowski J, Golusiński P, Więckowska B, Golusiński W. Oral cancer in young adults: should we approach these patients differently? *Front Oncol*. 2024;14:1297752. doi:10.3389/fonc.2024.1297752

45. Silva Monteiro L. Survival probabilities and trends for lip, oral cavity and oropharynx cancers in the Northern Region of Portugal in the period 2000–2009. *ecancermedicalscience*. 2018;12. doi:10.3332/ecancer.2018.855

46. Cariati P, Cabello-Serrano A, Perez-de Perceval-Tara M, Monsalve-Iglesias F, Martínez-Lara I. Oral and oropharyngeal squamous cell carcinoma in young adults: A retrospective study in Granada University Hospital. *Med Oral Patol Oral Cirugia Bucal*. 2017;22(6):e679-e685. doi:10.4317/medoral.21755

47. Martin-Granizo R, Rodriguez-Campo F, Naval L, Diaz Gonzalez FJ. Squamous cell carcinoma of the oral cavity in patients younger than 40 years. *Otolaryngol--Head Neck Surg Off J Am Acad Otolaryngol-Head Neck Surg*. 1997;117(3 Pt 1):268-275. doi:10.1016/s0194-5998(97)70185-2

48. Sasaki T, Moles DR, Imai Y, Speight PM. Clinico-pathological features of squamous cell carcinoma of the oral cavity in patients <40 years of age. *J Oral Pathol Med Off Publ Int Assoc Oral Pathol Am Acad Oral Pathol*. 2005;34(3):129-133. doi:10.1111/j.1600-0714.2004.00291.x

49. Hilly O, Shkedy Y, Hod R, et al. Carcinoma of the oral tongue in patients younger than 30 years: Comparison with patients older than 60 years. *Oral Oncol*. 2013;49(10):987-990. doi:10.1016/j.oraloncology.2013.07.005

50. Popovtzer A, Shpitzer T, Bahar G, Marshak G, Ulanovski D, Feinmesser R. Squamous Cell Carcinoma of the Oral Tongue in Young Patients. *The Laryngoscope*. 2004;114(5):915-917. doi:10.1097/00005537-200405000-00025

51. Soudry E, Preis M, Hod R, et al. Squamous cell carcinoma of the oral tongue in patients younger than 30 years: clinicopathologic features and outcome. *Clin Otolaryngol Off J ENT-UK Off J Neth Soc Oto-Rhino-Laryngol Cervico-Facial Surg*. 2010;35(4):307-312. doi:10.1111/j.1749-4486.2010.02164.x

52. Vered M, Dayan D, Dobriyan A, et al. Oral tongue squamous cell carcinoma: recurrent disease is associated with histopathologic risk score and young age. *J Cancer Res Clin Oncol*. 2010;136(7):1039-1048. doi:10.1007/s00432-009-0749-3

53. Yosefof E, Hilly O, Stern S, Bachar G, Shpitzer T, Mizrachi A. Squamous cell carcinoma of the oral tongue: Distinct epidemiological profile disease. *Head Neck*. 2020;42(9):2316-2320. doi:10.1002/hed.26177

54. Yosefof E, Tsur N, Zavdy O, et al. Prognostic Significance of Regional Disease in Young Patients with Oral Cancer: A Comparative Study. *The Laryngoscope*. 2024;134(5):2212-2220. doi:10.1002/lary.31187

55. Halboub E, Al-Mohaya M, Abdulhuq M, Al-Mandili A, Al-Anazi Y. Oral squamous cell carcinoma among Yemenis: Onset in young age and presentation at advanced stage. *J Clin Exp Dent*. 2012;4(4):e221-225. doi:10.4317/jced.50824

56. Tremblay S, Pintor Dos Reis P, Bradley G, et al. Young Patients With Oral Squamous Cell Carcinoma: Study of the Involvement of *GSTP1* and Deregulation of the Fanconi Anemia Genes. *Arch Otolaryngol Neck Surg*. 2006;132(9):958. doi:10.1001/archotol.132.9.958

57. Bommakanti KK, Abiri A, Han AY, Goshtasbi K, Kuan EC, St John MA. Stage‐Specific Survival in Young Patients With Oral Tongue Squamous Cell Carcinoma. *Otolaryngol Neck Surg*. 2023;168(3):398-406. doi:10.1177/01945998221101191

58. Campbell BR, Sanders CB, Netterville JL, et al. Early onset oral tongue squamous cell carcinoma: Associated factors and patient outcomes. *Head Neck*. 2019;41(6):1952-1960. doi:10.1002/hed.25650

59. Farhat MC, Dyalram D, Ord RA, Lubek JE. Oral squamous cell carcinoma in patients aged 45 and younger: Prognosis, survival, and quality of life. *Oral Surg Oral Med Oral Pathol Oral Radiol*. 2022;133(5):518-525. doi:10.1016/j.oooo.2021.08.023

60. Farquhar DR, Tanner AM, Masood MM, et al. Oral tongue carcinoma among young patients: An analysis of risk factors and survival. *Oral Oncol*. 2018;84:7-11. doi:10.1016/j.oraloncology.2018.06.014

61. Friedlander PL, Schantz SP, Shaha AR, Yu G, Shah JP. Squamous cell carcinoma of the tongue in young patients: a matched-pair analysis. *Head Neck*. 1998;20(5):363-368. doi:10.1002/(sici)1097-0347(199808)20:5%3C363::aid-hed1%3E3.0.co;2-w

62. Miller C, Shay A, Tajudeen B, et al. Clinical features and outcomes in young adults with oral tongue cancer. *Am J Otolaryngol*. 2019;40(1):93-96. doi:10.1016/j.amjoto.2018.09.022

63. Mneimneh WS, Xu B, Ghossein C, et al. Clinicopathologic Characteristics of Young Patients with Oral Squamous Cell Carcinoma. *Head Neck Pathol*. 2021;15(4):1099-1108. doi:10.1007/s12105-021-01320-w

64. Morris LGT, Patel SG, Shah JP, Ganly I. Squamous Cell Carcinoma of the Oral Tongue in the Pediatric Age Group: A Matched-Pair Analysis of Survival. *Arch Otolaryngol Neck Surg*. 2010;136(7):697. doi:10.1001/archoto.2010.94

65. Morris LGT, Ganly I. Outcomes of oral cavity squamous cell carcinoma in pediatric patients. *Oral Oncol*. 2010;46(4):292-296. doi:10.1016/j.oraloncology.2010.01.015

66. Mukdad L, Heineman TE, Alonso J, Badran KW, Kuan EC, St. John MA. Oral tongue squamous cell carcinoma survival as stratified by age and sex: A surveillance, epidemiology, and end results analysis. *The Laryngoscope*. 2019;129(9):2076-2081. doi:10.1002/lary.27720

67. Myers JN, Elkins T, Roberts D, Byers RM. Squamous cell carcinoma of the tongue in young adults: increasing incidence and factors that predict treatment outcomes. *Otolaryngol--Head Neck Surg Off J Am Acad Otolaryngol-Head Neck Surg*. 2000;122(1):44-51. doi:10.1016/S0194-5998(00)70142-2

68. Newman AN, Rice DH, Ossoff RH, Sisson GA. Carcinoma of the Tongue in Persons Younger Than 30 Years of Age. *Arch Otolaryngol - Head Neck Surg*. 1983;109(5):302-304. doi:10.1001/archotol.1983.00800190024006

69. Oliver JR, Wu SP, Chang CM, et al. Survival of oral tongue squamous cell carcinoma in young adults. *Head Neck*. 2019;41(9):2960-2968. doi:10.1002/hed.25772

70. Rodriguez N, Dickstein DR, Sindhu K, et al. Predictors of Treatment Failure in Young, Non-Smoking, Non-Drinking Patients with Squamous Cell Carcinoma of the Oral Tongue. *Int J Radiat Oncol*. 2024;118(5):e24. doi:10.1016/j.ijrobp.2024.01.057

71. Sturgis EM, Moore BA, Glisson BS, Kies MS, Shin DM, Byers RM. Neoadjuvant chemotherapy for squamous cell carcinoma of the oral tongue in young adults: A case series. *Head Neck*. 2005;27(9):748-756. doi:10.1002/hed.20240

72. Valero C, Yuan A, Zanoni DK, et al. Young non-smokers with oral cancer: What are we missing and why? *Oral Oncol*. 2022;127:105803. doi:10.1016/j.oraloncology.2022.105803

73. Satgunaseelan L, Porazinski S, Strbenac D, et al. Oral Squamous Cell Carcinoma in Young Patients Show Higher Rates of EGFR Amplification: Implications for Novel Personalized Therapy. *Front Oncol*. 2021;11:750852. doi:10.3389/fonc.2021.750852

74. Satgunaseelan L, Strbenac D, Willet C, et al. Whole genome duplication in oral squamous cell carcinoma in patients younger than 50 years: implications for prognosis and adverse clinicopathological factors. *Genes Chromosomes Cancer*. 2022;61(9):561-571. doi:10.1002/gcc.23076

75. Amaral MGD, Sena LSBD, Batista AC, et al. FoxP3+ regulatory T cells in oral tongue squamous cell carcinoma in young and older patients. *Braz Oral Res*. 2020;34:e096. doi:10.1590/1807-3107bor-2020.vol34.0096

76. Barnabé LÉG, Batista AC, Mendonça EFD, Nonaka CFW, Alves PM. Cell cycle markers and apoptotic proteins in oral tongue squamous cell carcinoma in young and elderly patients. *Braz Oral Res*. 2019;33:e103. doi:10.1590/1807-3107bor-2019.vol33.0103

77. Borba Ribeiro KR, Lira Júnior C, Marinho SA, De Carvalho SHG, Agripino GG, Sarmento DJDS. Epidemiological profile of young patients with squamous cell carcinoma in northeast Brazil. *J Investig Clin Dent*. 2019;10(4):e12436. doi:10.1111/jicd.12436

78. Cury SS, Miranda PMD, Marchi FA, et al. Germline variants in DNA repair genes are associated with young-onset head and neck cancer. *Oral Oncol*. 2021;122:105545. doi:10.1016/j.oraloncology.2021.105545

79. Amorim MDM, Pires ALPV, Assis ALMDS, Silva CALD, Santos JND, Freitas VS. Survival of young and elderly adults with oral squamous cell carcinoma in a population in northeastern Brazil. *Braz J Oral Sci*. 2023;22:e230008. doi:10.20396/bjos.v22i00.8670008

80. Frare JC, Sawazaki-Calone I, Ayroza-Rangel ALC, et al. Histopathological grading systems analysis of oral squamous cell carcinomas of young patients. *Med Oral Patol Oral Cirugia Bucal*. 2016;21(3):e285-298. doi:10.4317/medoral.20953

81. Hirota SK, Braga FPF, Penha SS, Sugaya NN, Migliari DA. Risk factors for oral squamous cell carcinoma in young and older Brazilian patients: a comparative analysis. *Med Oral Patol Oral Cirugia Bucal*. 2008;13(4):E227-231.

82. Kaminagakura E, Vartanian JG, Da Silva SD, Dos Santos CR, Kowalski LP. Case‐control study on prognostic factors in oral squamous cell carcinoma in young patients. *Head Neck*. 2010;32(11):1460-1466. doi:10.1002/hed.21347

83. Ribeiro ACP, Silva ARS, Simonato LE, Salzedas LMP, Sundefeld MLMM, Soubhia AMP. Clinical and histopathological analysis of oral squamous cell carcinoma in young people: a descriptive study in Brazilians. *Br J Oral Maxillofac Surg*. 2009;47(2):95-98. doi:10.1016/j.bjoms.2008.05.004

84. Santos HB de P, dos Santos TKG, Paz AR, et al. Clinical findings and risk factors to oral squamous cell carcinoma in young patients: A 12-year retrospective analysis. *Med Oral Patol Oral Cirugia Bucal*. 2016;21(2):e151-156. doi:10.4317/medoral.20770

85. Tomo S, Neto SC, Collado FU, et al. Head and neck squamous cell carcinoma in young patients: a 26-year clinicopathologic retrospective study in a Brazilian specialized center. *Med Oral Patol Oral Cirugia Bucal*. 2020;25(3):e416-e424. doi:10.4317/medoral.23461
